# Comparison of calibration strategies for accurate quantitation by isotope dilution mass spectrometry: a case study of ochratoxin A in flour

**DOI:** 10.1007/s00216-023-05053-3

**Published:** 2023-12-04

**Authors:** Jennifer Bates, Adilah Bahadoor, Sheryl A. Tittlemier, Jeremy E. Melanson

**Affiliations:** 1 National Research Council Canada, Metrology, 1200 Montreal Road, Ottawa, ON K1A 0R6 Canada; 2 Canadian Grain Commission, Grain Research Laboratory, 1404-303 Main Street, Winnipeg, MB R3C 3G8 Canada

**Keywords:** Mycotoxins, Internal standard, Liquid chromatography-mass spectrometry, Trace contaminants in food

## Abstract

Analysis of low-level organic contaminants in complex matrices is essential for monitoring global food safety. However, balancing sample throughput with complex experimental designs and/or sample clean-up to best reduce matrix effects is a constant challenge. Multiple strategies exist to mitigate these effects, with internal standard-based methods such as isotope dilution mass spectrometry (IDMS) being the most advantageous. Here, multiple internal calibration strategies were investigated for the quantification of ochratoxin A (OTA) in wheat samples by liquid chromatography-mass spectrometry (LC-MS). Internal standard-based quantitation methods such as single (ID^1^MS), double (ID^2^MS), and quintuple (ID^5^MS) isotope dilution mass spectrometry, as well as external standard calibration, were explored and compared. A certified reference material (CRM) of OTA in flour, MYCO-1, was used to evaluate the accuracy of each method. External calibration generated results 18–38% lower than the certified value for MYCO-1, largely due to matrix suppression effects. Concurrently, consistently lower OTA mass fractions were obtained for the wheat samples upon quantitation by external calibration as opposed to ID^1^MS, ID^2^MS, and ID^5^MS. All isotope dilution methods produced results that fell within the expected range for MYCO-1 (3.17–4.93 µg/kg), validating their accuracy. However, an average 6% decrease in the OTA mass fraction was observed from results obtained by ID^1^MS compared to those by ID^2^MS and ID^5^MS. Upon scrutiny, these differences were attributed to an isotopic enrichment bias in the isotopically labelled internal standard [^13^C_6_]-OTA that was used for ID^1^MS, the OTAL-1 CRM. The advantages and limitations of each isotopic method are illustrated.

## Introduction

Trace analysis of organic contaminants is vital for environmental monitoring and ensuring food safety. While modern liquid chromatography-mass spectrometry (LC-MS) instruments can readily detect many low-level contaminants, especially in pure solutions, accurate quantitation in complex matrices remains challenging. Ionization suppression, or in rare cases ionization enhancement, of trace analytes by co-eluting high-abundance matrix components is a well-known phenomenon that can bias results [1–5]. The soft nature of electrospray ionization (ESI) that allows for the generation of intact molecular ions, which is typically ESI’s greatest advantage, also limits its quantitative performance. Therefore, matrix effects due to ionization suppression need to be assessed to ensure accuracy of LC-MS methods.

Several strategies exist for mitigating the effects of ionization suppression [2, 6–9]. Additional sample clean-up steps can reduce ionization suppression in some cases, but this can sometimes result in a loss of analyte, further biasing results. Standard addition methods can overcome matrix effects such as ionization suppression, but this can be labor intensive and generally results in poor method precision. Matrix-matched calibration can be effective at mitigating ionization suppression [1, 10–14], but it relies on the availability of a blank matrix perfectly matched to the sample, which is not always available. Arguably the best way to circumvent matrix effects is with the use of an internal standard [15]. The internal standard should be added prior to extraction to equilibrate with the analyte and to compensate for any losses [16]. The internal standard should be as structurally similar as possible to the analyte to achieve perfect co-elution and equal ionization efficiency, requirements easily fulfilled by an isotopic derivative. Ideally, an increase of at least three mass units is ideal to avoid overlap of the signals between the native and the isotopically labelled internal standard [15]. Isotopic derivatives are typically generated synthetically via the incorporation of an appropriate number of deuterium or ^13^C atoms [15]. Alternatively, biosynthetic labelling with micro-organisms such as fungi, bacteria, or algae cultured in isotopically enriched media can yield fully labelled analogues [17].

Internal standards can be used in various ways for calibration in LC-MS [18]. In the simplest case, referred to as single isotope dilution mass spectrometry (ID^1^MS) below, a known amount of internal standard is spiked into the sample. Given the concentration of the internal standard is known, the amount of analyte is then determined from a simple ratio of the analytical signals from the analyte and internal standard. However, isotopically enriched standards available as reference standards or certified reference materials (CRMs) are rare. Double isotope dilution mass spectrometry (ID^2^MS) can be employed, whereby the internal standard is spiked into both the sample and the corresponding unlabelled reference standard (calibration standard solution), which negates the need to know the concentration of the internal standard. Further, many additional sources of errors can be negated by using “exact-matching” ID^2^MS, where an iterative process is undertaken to match the ratios of labelled to unlabelled analyte measured in both the calibration standard solution and samples [19]. Finally, any deviations from perfect exact-matching can be overcome with a “multi-spike” approach where multiple calibration standard solutions bracket the samples. The analyte concentration can then be determined graphically or with complex equations [18, 20, 21].

While ID^2^MS or ID^n^MS have yielded high-accuracy results in previous studies, both require additional steps to prepare either one (for ID^2^MS) or several (for ID^n^MS) calibration standard solutions [18]. Arguably, ID^1^MS is the simplest approach since only one sample extract (sample spiked with a labelled reference standard) is required and no calibration standard solutions are needed. To compare the relative strengths and weaknesses of ID^1^MS, ID^2^MS, and ID^5^MS, an unlabelled ochratoxin A CRM (OTAN-1) and/or a [^13^C_6_]-ochratoxin A CRM (OTAL-1) was used to quantitate OTA in Canada Western Red Spring (CWRS) and Canada Western Amber Durum (CWAD) wheat samples, with the rye flour matrix CRM MYCO-1 as the quality control sample. External calibration was also performed to provide a baseline to which the advanced calibration strategies could be compared.

This report compares various calibration strategies for the quantitation of OTA in flour. The accuracy of each approach will be discussed, along with the practical considerations such as the level of complexity and sample throughput. Finally, bias in quantitation results caused by differences in isotopic composition between the analyte and internal standard when employing simple calibration strategies will be highlighted.

## Experimental

### Materials and reagents

Four ground samples of each Canada Western Red Spring (CWRS) and Canada Western Amber Durum (CWAD) wheat were obtained from the Canadian Grain Commission (Winnipeg MB, Canada). Acetonitrile (Optima® grade), glacial acetic acid (HPLC grade), and formic acid (Optima® LC/MS grade) were obtained from Fisher Scientific (Nepean ON, Canada). Ultrapure water (18.2 MΩ cm) was produced in-house with a Millipore Milli-Q Reference A+ Ultrapure water purification system. Certified reference materials (CRMs) consisting of solutions of native OTA (OTAN-1), stable isotope labelled [^13^C_6_]-OTA (OTAL-1), and the rye flour MYCO-1 were produced by the National Research Council of Canada (NRC). MYCO-1 was value assigned by exact-matching ID^2^MS [22, 23], and served as a quality control sample to evaluate the accuracy of each calibration method described below.

### Calibration standard solutions preparation

An internal standard solution and native standard solution were gravimetrically prepared at a target level of 32 ng/g using OTAL-1 and OTAN-1, respectively. In order to minimize the risk of adhesion of OTA onto glass surfaces, solutions were prepared in silanized amber glass vials using acetonitrile with 0.1% formic acid. Vials contained caps with rubber septa for syringe sampling.

Four calibration standard solutions (Cals) were gravimetrically prepared using separate gas-tight syringes with 0.036 g, 0.082 g, 0.39 g, or 0.78 g (~40 µL, 100 µL, 500 µL, and 1000 µL respectively) of the OTA native standard solution and 0.39 g (~500 µL) of the [^13^C_6_]-OTA internal standard solution. The extraction solvent, 85% acetonitrile/water (by volume), was added gravimetrically to achieve a total solution mass of ~11.5 g. A sub-sample of each calibration standard solution was transferred, without further dilution, to a silanized amber HPLC vial for analysis.

### Sample extract preparation

The samples of Canada Western Red Spring (CWRS) and Canada Western Amber Durum (CWAD) wheat obtained from the Canadian Grain Commission are representative of randomly selected shipments of each cereal grain [24]. The shipments were prepared through a detailed sampling procedure which included grinding, sub-sampling, and homogeniziation [25]. The sample preparation and analysis of all CWRS and CWAD samples described herein took place over 3 days. Each day, three independent test portions from different CWRS and/or CWAD samples were extracted. Additionally, three independent test portions from a bottle of the CRM MYCO-1 were extracted to serve as quality control samples.

Extraction of ochratoxin A from the flour samples was a slightly modified version of the validated method described by Bates et al. [23]. All sample extracts were gravimetrically prepared with 5 g of flour, 0.39 g of the internal standard solution (~500 µL, gas-tight syringe), and 11.1 g of 85% acetonitrile/water (by volume, ~13 mL). The samples were vortexed, placed on the orbital shaker for 1 h at maximum speed (450–475 RPM), and then centrifuged at 7200 RPM for 10 min. A sub-sample of the extract was transferred, without further dilution, to a silanized amber HPLC vial for analysis. The same sample extracts were used regardless of the calibration strategy employed for the quantitation of ochratoxin A.

### LC-high-resolution MS (HRMS)

Analyses were performed in a very similar manner to the LC-HRMS method described by Bates et al. [23]. A Vanquish UHPLC (Thermo Fisher Scientific), used for chromatographic separation, was interfaced with a heated electrospray source of an Orbitrap Fusion Lumos Tribrid mass spectrometer (Thermo Fisher Scientific) operating in positive ion mode. Calibration standard solutions were analyzed in quadruplicate to bracket extracted samples which were analyzed in triplicate. Samples were injected onto an Agilent ZORBAX Eclipse Plus C18 column (2.1×150 mm, 3.5 µm, 95 Å) using an injection volume of 4 µL. Elution proceeded at 0.3 mL/min with mobile phases consisting of 0.05% acetic acid in (A) water and (B) acetonitrile with the column temperature controlled at 25 °C. The separation was achieved using a gradient as follows: 0–10 min (30 to 60% B), 10–10.1 min (60 to 95% B), 10.1–16 min (95% B), 16–16.1 min (95 to 30% B), and 16.1–22 min (30% B). Data was acquired by scanning from *m/z* 375–425 at 120,000 mass resolution. The spray needle was kept at a potential of 3.9 kV while the ion transfer tube temperature was set to 300 °C and the vaporizer to 250 °C. The sheath gas was set to 40, auxiliary gas to 15, and the sweep gas to 1, all in arbitrary units. For quantitation, peak areas were determined from extracted ≤10 ppm *m/z* windows around the exact monoisotopic masses of [M+H]^+^ for ochratoxin A at 404.08954 Da and [^13^C_6_]-ochratoxin A at 410.10967 Da.

## Results and discussion

In many matrices, ochratoxin A is present at low levels (µg/kg) making precise and accurate analysis difficult. To add to this challenge, OTA is known to be heterogeneously dispersed in many bulk commodities such as raw grains [26, 27]. Although steps can be taken to mitigate heterogeneity, such as in the preparation of the CRM MYCO-1, it cannot be completely eliminated [23]. Therefore, the results obtained for each quantitation method described herein were calculated using this same set of extracted samples to eliminate any homogeneity issues as a potential source for any observed differences allowing for the direct comparison of each calibration strategy.

### External standard calibration

Official AOAC methods and European Standards exist for the quantitation of ochratoxin A in a variety of different matrices including coffee, barley, wine, and beer [28–34]. These methods use liquid chromatography with fluorescence detection (LC-FLD) and require preparation of a calibration curve with ochratoxin A standard solutions [25, 35, 36]. These external standard calibration methods require complete extraction of the analyte or the results must be recovery-corrected. The percent recovery must be determined from an independent experiment typically using a blank matrix, which as previously mentioned is not always available. Additionally, a clean-up step is usually required such as an immunoaffinity separation. Use of LC-MS methods compared to LC-FLD increases sensitivity and selectivity, proving valuable for OTA analysis at trace levels. However, matrix interferences can be problematic, and for external calibration methods, sample clean-up may still be required [37, 38].

**Fig. 1:**
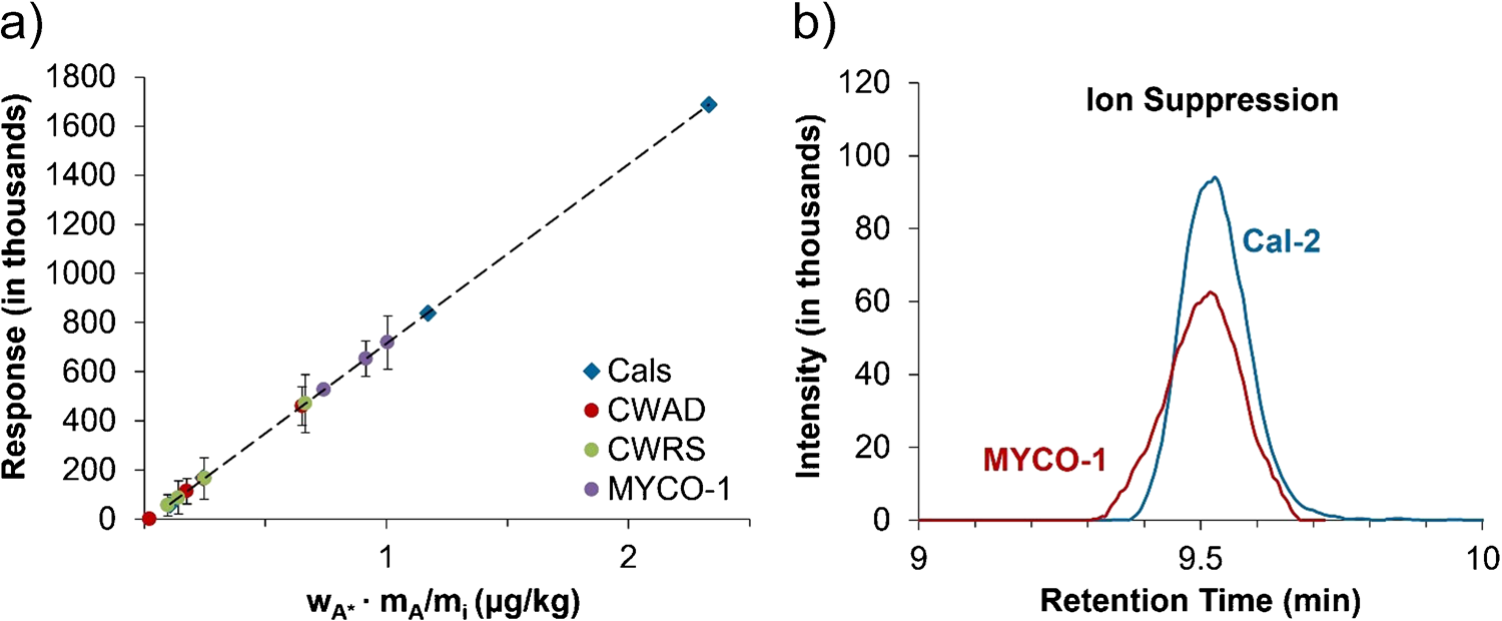
**a** External standard calibration curve with results of each sample (average of triplicate extractions). The error bars represent the standard deviation of the triplicate extractions and reflect the heterogeneity of the samples. All samples shown on the same calibration curve for simplicity. **b** LC-HRMS chromatograms of OTA in calibration standard solution 2 (Cal-2, 1.2 ng/g) and MYCO-1 extract (1.3 ng/g), revealing ion suppression

The mass fraction of OTA was calculated for all flour samples using external standard calibration. Preliminary work to determine the appropriate calibration range was performed by comparing the CWRS and CWAD samples to MYCO-1. The calibration curve was prepared by gravimetrically diluting the native standard solution containing the CRM OTAN-1 in the extraction solvent, 85% acetonitrile/water (by volume), to obtain a concentration range of 0.1–2.5 ng/g. Results are therefore traceable through OTAN-1. All sample extracts and calibration standard solutions (Cals) were analyzed as is, with no sample clean-up, by LC-HRMS to determine peak areas. The calibration curve was fitted using a linear function and the resulting equation solved for each sample to determine the mass fraction of OTA. As shown in Fig. 1a, the peak area for each sample fell toward the bottom of the calibration curve. The mass fraction of OTA in quality control samples of MYCO-1 was 18–38% lower than the certified value of 4.05 ± 0.88 µg/kg, *k* = 2 (MYCO-1 samples A–C in Table 1). Two out of the three samples even fell outside the certified uncertainty range with values of 2.91 and 2.50 µg/kg for the mass fraction of OTA by external calibration. As shown in Fig. 1b, an overlay of the chromatograms for calibration standard solution 2 (Cal-2, 1.2 ng/g) and MYCO-1 extract (1.3 ng/g) reveals significant ion suppression in the extracted sample. Evidence of ion suppression, combined with the poor results obtained for quality control samples of MYCO-1, indicates external calibration is not an ideal method for the quantitation of OTA in flour. It is therefore unlikely that the OTA values in CWRS and CWAD samples (Table 1) are an accurate representation of the true value.

**Table 1:** The mass fraction of OTA in various flour samples obtained by external standard calibration, based on the average of triplicate extractions (*N.D.*, not detected). The uncertainty associated with the mass fraction (*u*) is the average measurement uncertainty which includes contributions from the CRM OTAN-1 and triplicate analysis of the same extract

	*w*_A_, µg/kg	*u*, µg/kg
CWAD-1	0.82	0.03
CWAD-2	N.D.	N.D.
CWAD-3	0.56	0.04
CWAD-4	2.09	0.07
CWRS-1	2.23	0.07
CWRS-2	0.84	0.05
CWRS-3	0.48	0.03
CWRS-4	0.32	0.01
MYCO-1 A	2.91	0.09
MYCO-1 B	3.32	0.10
MYCO-1 C	2.50	0.07

### Isotope dilution methods

Isotope dilution methods include the addition of an isotopically enriched compound to the sample to counteract matrix effects. Traceable quantitation can then be achieved through the natural reference standard with use of an internal standard as a control or it can be obtained directly through the internal standard, if the latter is a reference material or a CRM. Numerous labelled derivatives are available for mycotoxins; however, their concentration is not always known with confidence and they lack traceability. OTAN-1 and OTAL-1 are both CRMs produced in accordance with ISO 17034:2016 [39] with a certified mass fraction of OTA (11.03 ± 0.32 µg/g, *k* =2) or [^13^C_6_]-OTA (4.91 ± 0.16 µg/g, *k* = 2) respectively [40, 41]. They can be used for the quantitation of OTA in a wide range of matrices by employing isotope dilution strategies. An advantage of isotope dilution over external standard calibration is that complete analyte extraction may not be required, but rather complete equilibration between the analyte and internal standard must be achieved [42]. If the internal standard is added prior to extraction and complete isotopic equilibrium is reached, it is assumed that any loss of analyte will be compensated for even if the extraction is incomplete [16]. Additionally, as ratios of native analyte peak area to stable isotope-labelled internal standard peak area are used instead of absolute peak areas, solvent evaporation of samples is no longer an utmost concern. Calibration standard solutions (Cals) can then be analyzed multiple times, even days later, provided the analyte is stable.

### Single isotope dilution mass spectrometry (ID^1^MS)

**Fig. 2:**
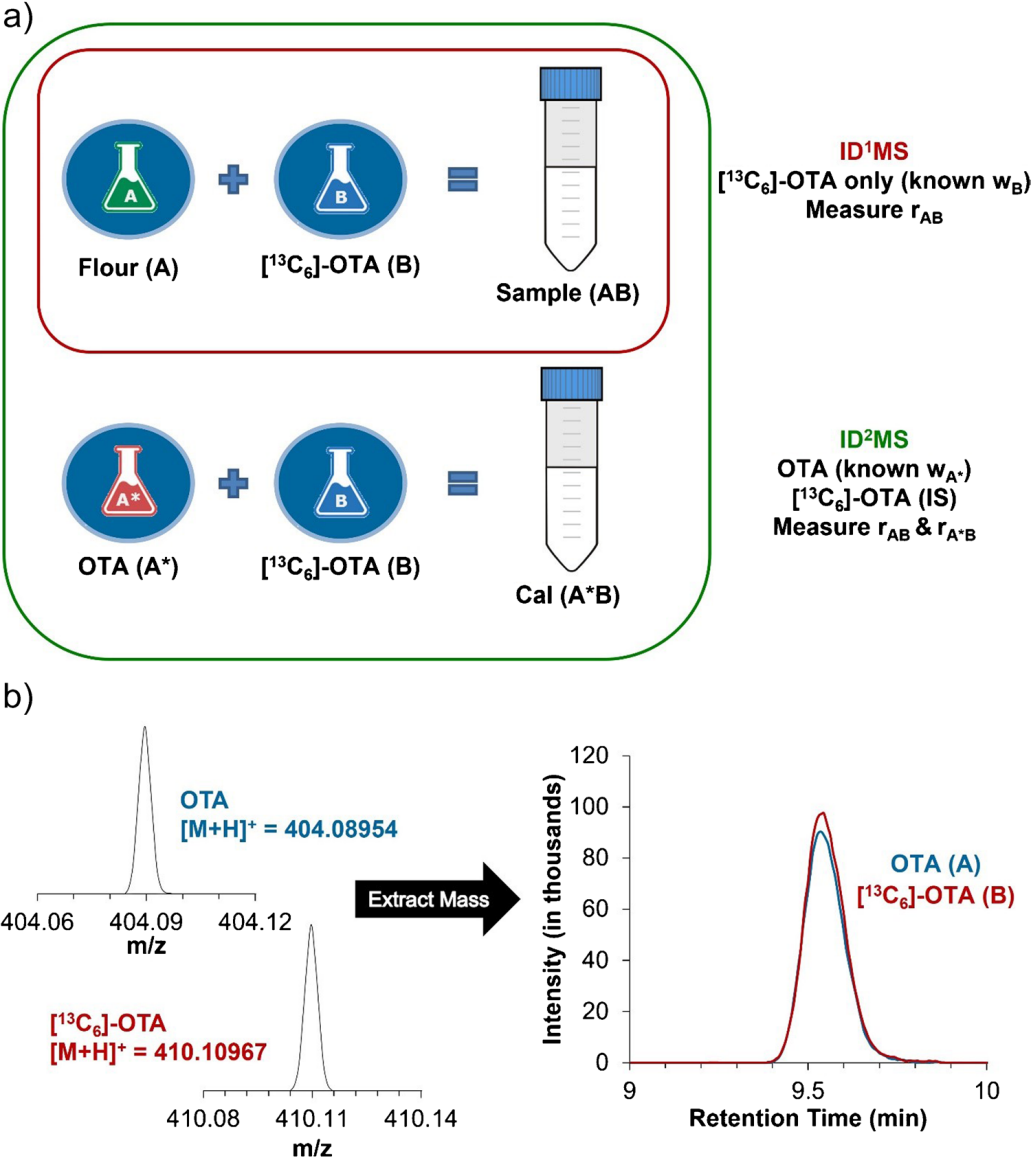
**a** Experimental scheme for ID^1^MS (sample) and ID^2^MS (sample and calibration standard solution). OTA (A*) comes from the native working solution of diluted OTAN-1 and [^13^C_6_]-OTA (B) comes from the internal standard solution of diluted OTAL-1. **b** Unlabelled OTA and [^13^C_6_]-OTA exhibit similar elution and ionization profiles by LC-MS

When quantitation is achieved directly through the internal standard, this is known as single isotope dilution mass spectrometry (ID^1^MS). It requires only an isotopically enriched standard to act as the primary calibrator, providing an advantage over higher order isotope dilution (ID^n^MS) methods due to its simplicity. Additionally, only a single sample is required, reducing sample preparation and analysis time as well as eliminating the need for any calibration standard solutions (Fig. 2a). To illustrate its effectiveness, the mass fraction of OTA in CWRS and CWAD wheat samples, as well as quality control samples of MYCO-1, was determined by ID^1^MS using OTAL-1 as the internal calibrant. As shown in Fig. 2b, both unlabelled OTA and [^13^C_6_]-OTA have similar elution and ionization profiles by LC-MS. Therefore, OTAL-1 is expected to reliably provide very accurate measurement values for OTA when used as a calibrant.

Each sample was spiked with known amounts of the internal standard solution containing the CRM OTAL-1 and analyzed by LC-HRMS. Spectral windows centered at the exact monoisotopic masses of 404.08954 Da for OTA and 410.10967 Da for [^13^C_6_]-OTA were extracted and the ratio of peak areas of the two isotopic forms of ochratoxin A was measured in each sample (unlabelled:labelled). The mass fraction of OTA (*w*_*A*_) in each sample was calculated as follows:

1 $${w}_{\mathrm{A}}={w}_{\mathrm{B}}\bullet \frac{{r}_{\mathrm{B}}-{r}_{\mathrm{AB}}}{{r}_{\mathrm{AB}}-{r}_{\mathrm{A}}}\bullet \frac{{m}_{\mathrm{B}\left(\mathrm{AB}\right)}}{{m}_{\mathrm{A}\left(\mathrm{AB}\right)}}\bullet \frac{{M}_{A}}{{M}_{B}}$$

where *w*_B_ is the mass fraction of [^13^C_6_]-OTA in the internal standard solution, *r*_A_ is the isotope ratio of OTA in the sample, *r*_B_ is the isotope ratio of [^13^C_6_]-OTA in the internal standard solution, *r*_AB_ is the isotope ratio in the sample extract (AB), *m*_B(AB)_ is the mass of [^13^C_6_]-OTA in the sample, *m*_A(AB)_ is the mass of flour in the sample, *M*_A_ is the molar mass of OTA, and *M*_B_ is the molar mass of [^13^C_6_]-OTA. Results for quality control samples of MYCO-1 were in agreement with the certified value of OTA, 4.05 ± 0.88 µg/kg,* k* = 2 (Table 2).

**Table 2:** The mass fraction of OTA in various flour samples obtained by each quantitation method, based on the average of triplicate extractions (*N.D.*, not detected). The uncertainty (*u*) is the average measurement uncertainty which includes contributions from the CRMs OTAN-1 or OTAL-1

	ID^1^MS		ID^2^MS		ID^5^MS	
	*w*_A_, µg/kg	*u*, µg/kg	*w*_A_, µg/kg	*u*, µg/kg	*w*_A_, µg/kg	*u*, µg/kg
CWAD-1	1.31	0.06	1.39	0.07	1.38	0.07
CWAD-2	N.D.	N.D.	N.D.	N.D.	N.D.	N.D.
CWAD-3	0.83	0.06	0.89	0.06	0.89	0.07
CWAD-4	3.04	0.12	3.21	0.13	3.20	0.13
CWRS-1	3.69	0.12	3.90	0.13	3.90	0.13
CWRS-2	1.34	0.06	1.43	0.06	1.42	0.06
CWRS-3	0.73	0.04	0.77	0.04	0.78	0.04
CWRS-4	0.45	0.02	0.49	0.03	0.48	0.03
MYCO-1 A	3.72	0.08	3.92	0.09	3.91	0.09
MYCO-1 B	4.33	0.12	4.54	0.13	4.54	0.13
MYCO-1 C	3.57	0.15	3.77	0.16	3.78	0.16

### Double isotope dilution mass spectrometry (ID^2^MS)

As previously mentioned, if the exact concentration of the labelled compound is unknown or lacks the appropriate traceability, quantitation cannot be obtained by ID^1^MS. Alternatively, single-point calibration with internal standard known as double isotope dilution mass spectrometry (ID^2^MS) can be used. With this method, OTAN-1 and OTAL-1 serve as a primary calibrator and internal standard respectively, and *w*_B_ (the mass fraction of OTAL-1) is no longer required. Samples were prepared, same as those for ID^1^MS, consisting of flour spiked with known amounts of the internal working solution containing the CRM OTAL-1 ([^13^C_6_]-OTA). Additionally, a calibration standard solution containing a known amount of the native standard solution (unlabelled OTA from OTAN-1) and internal standard solution ([^13^C_6_]-OTA from OTAL-1) was prepared (Fig. 2). The ratio of OTA to [^13^C_6_]-OTA in each sample extract and calibration standard solution (Cal) was measured using LC-HRMS and the mass fraction of ochratoxin A was then calculated according to the standard ID^2^MS equation [18] as follows:

2 $${w}_{\mathrm{A}}={w}_{\mathrm{A}*}\bullet \frac{{r}_{\mathrm{A}*}-{r}_{{\mathrm{A}}^{*}\mathrm{B}}}{{r}_{{\mathrm{A}}^{*}\mathrm{B}}-{r}_{\mathrm{B}}}\bullet \frac{{r}_{\mathrm{B}}-{r}_{\mathrm{AB}}}{{r}_{\mathrm{AB}}-{r}_{\mathrm{A}}}\bullet \frac{{m}_{{\mathrm{A}}^{*}\left({\mathrm{A}}^{*}\mathrm{B}\right)}}{{m}_{\mathrm{B}\left({\mathrm{A}}^{*}\mathrm{B}\right)}}\bullet \frac{{m}_{\mathrm{B}\left(\mathrm{AB}\right)}}{{m}_{\mathrm{A}\left(\mathrm{AB}\right)}}$$

where *w*_A*_ is the mass fraction of OTA in the native standard solution, *r*_A_ is the isotope ratio of OTA in the sample, *r*_A*_ is the isotope ratio of OTA in the native standard solution (we assume *r*_A_ = *r*_A*_), *r*_B_ is the isotope ratio of [^13^C_6_]-OTA in the internal standard solution, *r*_A*B_ is the isotope ratio in the calibration standard solution (A*B), *r*_AB_ is the isotope ratio in the sample (AB), *m*_A*(A*B)_ is the mass of OTA in the calibration standard solution, *m*_B(A*B)_ is the mass of [^13^C_6_]-OTA in the calibration standard solution, *m*_B(AB)_ is the mass of [^13^C_6_]-OTA in the sample, and *m*_A(AB)_ is the mass of flour in the sample. The mass fraction of OTA for each sample was calculated using the calibration standard solution (Cal) which had an isotope ratio (*r*_A*B_) closest to that of the sample extract (*r*_AB_). Again, the results for quality control samples of MYCO-1 fell within the certified range (Table 2).

A similar method, known as exact-matching ID^2^MS, was used to certify the mass fraction of OTA in MYCO-1 [22, 23]. For exact-matching, the concentration of the unlabelled reference standard in the calibration standard solution (Cal) should match the estimated mass fraction of the analyte in the sample; then, both are spiked with equal amounts of internal standard (*m*_B(A*B)_ = *m*_B(AB)_). Additionally, both sample and calibration standard solution are prepared such that the signal ratio of unlabelled to labelled is as close to 1:1 as possible (*r*_A*B_ = *r*_AB_ = 1). This method minimizes mass bias effects ultimately providing a more accurate result and reducing overall uncertainties [19]. Exact-matching ID^2^MS can prove challenging as initial knowledge about the concentration of analyte is necessary. Preliminary work is required to first estimate the mass fraction of analyte in each sample. Once known, the concentration of the unlabelled reference standard in the calibration standard solution can be determined and the ideal ratio (1:1) can be fully optimized. Additionally, as each sample will likely differ in the amount of analyte present, an independent calibration standard solution would need to be prepared for each sample. Although this approach can yield highly accurate results, it is very labor intensive and thus unsuitable for high-throughput analysis. Therefore, exact-matching ID^2^MS was not used to quantitate OTA in CWRS and CWAD samples.

### Higher order isotope dilution mass spectrometry (ID^n^MS)

**Fig. 3:**
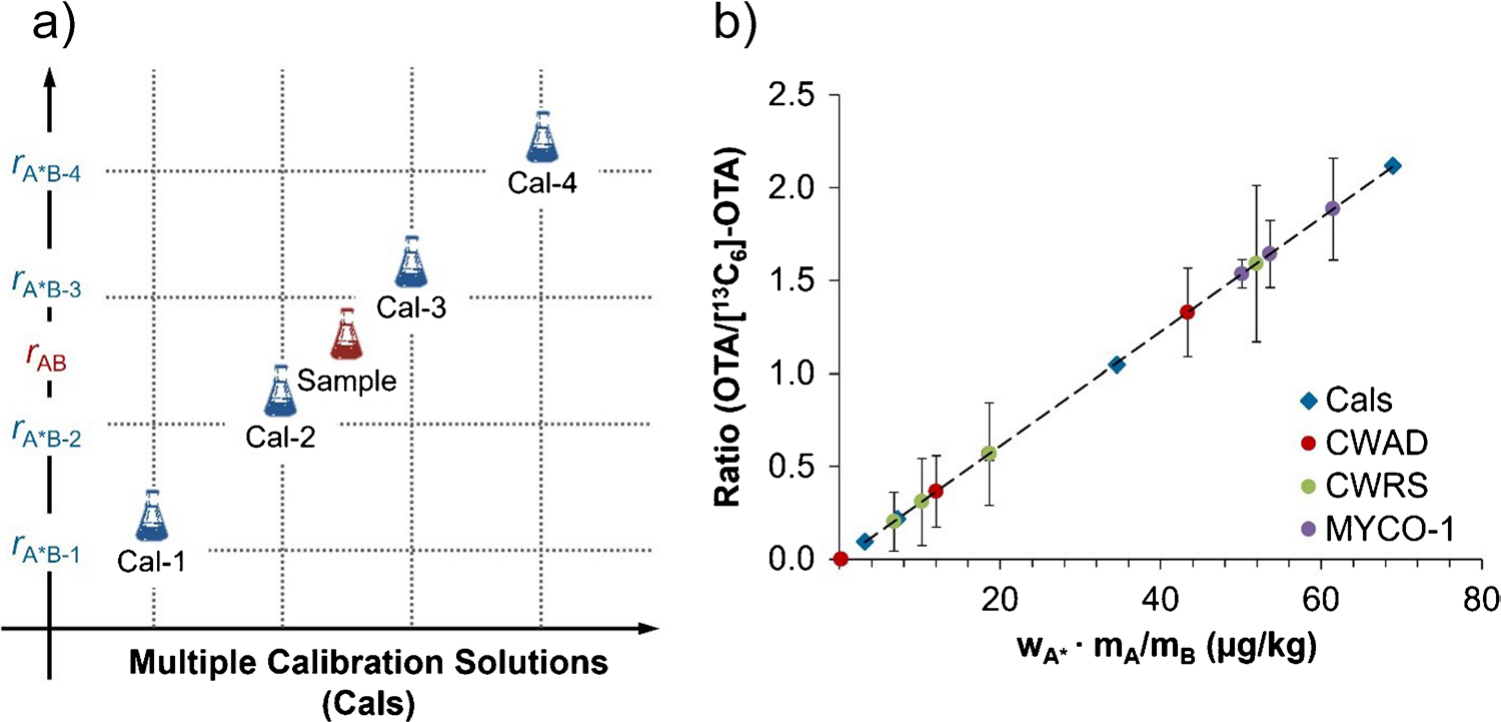
**a** Experimental scheme for quintuple isotope dilution mass spectrometry (ID^5^MS) including four calibration standard solutions (Cal-1 to Cal-4) with different isotope ratios shown to bracket the isotope ratio in the sample (AB). **b** Isotope ratio calibration curve with results of each sample (average of triplicate extractions). The error bars represent the standard deviation of the triplicate extractions and reflect the heterogeneity of the samples. All samples shown on the same calibration curve for simplicity

An alternate approach, ideal for measuring samples over a wider range of concentrations, employs multiple calibration standard solutions (Cals) of the unlabelled reference standard and internal standard across a calibration range (Fig. 3a). Depending on the number of calibration standard solutions prepared, this method can be referred to as triple (ID^3^MS, 2 calibration standard solutions), quadruple (ID^4^MS, 3 calibration standard solutions), and quintuple (ID^5^MS, 4 calibration standard solutions) isotope dilution mass spectrometry and so forth. The resulting mass fraction can be determined using a specific isotope dilution equation [18] or a calibration curve can be fitted similar to external standard calibration. However, rather than plotting peak areas, ratios of peak areas of native to labelled isotopes are used instead to mitigate matrix effects.

For the quantitation of OTA in the CWAD and CWRS samples, the ID^5^MS method was used. Four calibration standard solutions (Cals) were gravimetrically prepared using varying amounts of the native standard solution, containing the CRM OTAN-1, and a similar amount of internal standard solution, containing the CRM OTAL-1, to obtain isotope ratios of OTA/[^13^C_6_]-OTA at ~0.08:1, 0.2:1, 1:1, and 2:1. The internal standard solution added to the calibration standard solutions was equal to the amount spiked in the sample extracts. All samples were analyzed by LC-HRMS and using the peak area ratio of OTA to [^13^C_6_]-OTA, a calibration curve was fitted using a linear function (Fig. 3b). While a linear model is commonly applied for routine use and adequate in many cases, we note that the theoretical shape of the calibration curve is inherently nonlinear [21]. The calibration curve equation was solved for each sample with the mass fraction of OTA in quality control samples of MYCO-1 falling within the certified range (Table 2). This approach can be beneficial for samples where the levels of OTA are unknown, as well as when samples contain a wide range of differing amounts of analyte. Although this method may require multiple calibration standard solutions, the calibration curve range can be tailored to include as many or as few points as desired. If the isotope ratio in a given sample extract falls outside of the calibration range, additional calibration standard solutions can be added and the samples re-analyzed. Provided the analyte is stable, a single calibration curve can be re-analyzed multiple times allowing the analysis of a large number of samples, maximizing throughput.

### Method comparison

**Fig. 4:**
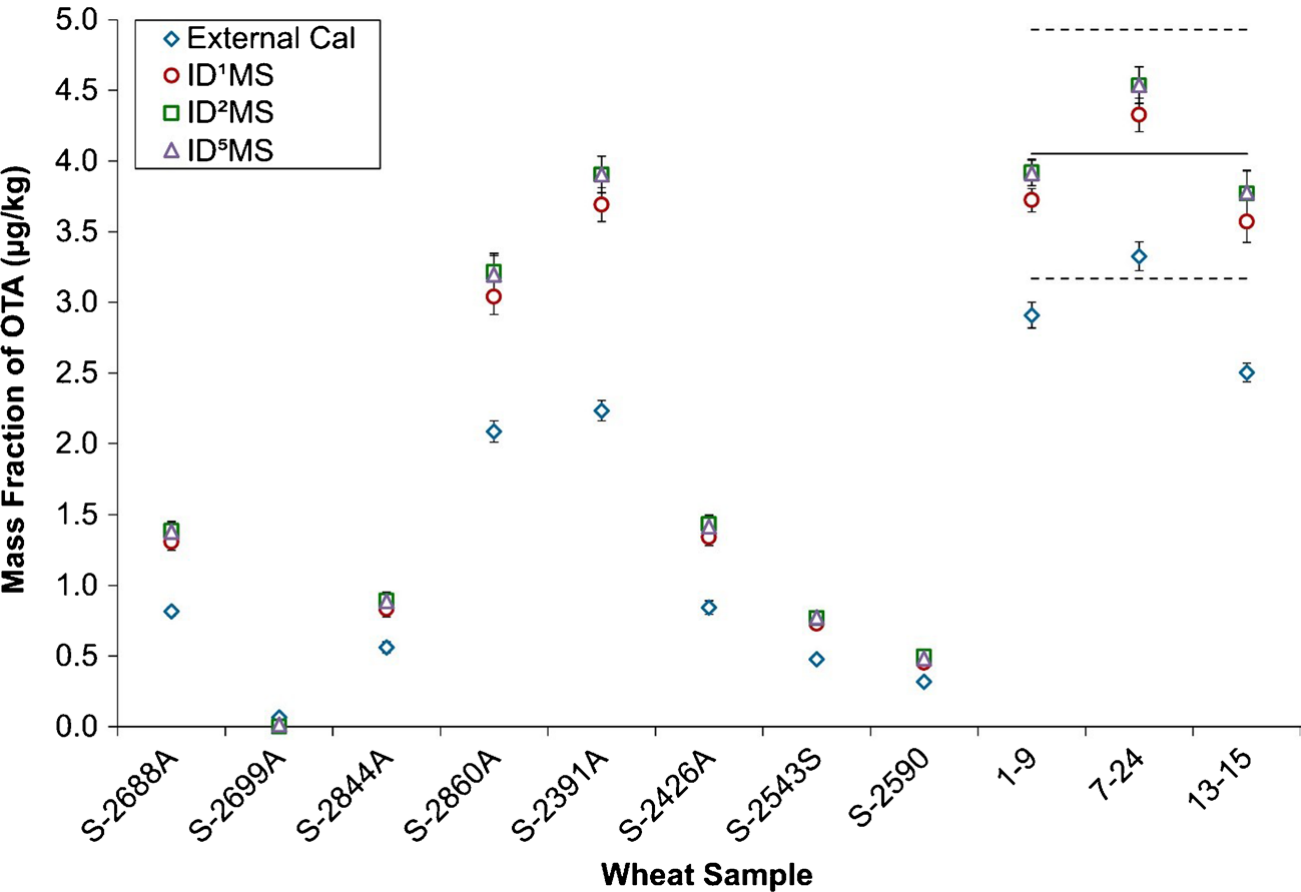
Mass fraction of OTA obtained for each quantitation method (average of triplicate extractions with an average measurement uncertainty). The solid line represents the certified value for OTA in MYCO-1 and the dotted lines represent the expanded uncertainty

The final mass fractions of OTA obtained from each calibration method, external standard calibration, and single (ID^1^MS), double (ID^2^MS), and quintuple (ID^5^MS) isotope dilution mass spectrometry are shown in Fig. 4. For external calibration, the mass fraction of OTA obtained for each sample was much lower than those obtained by isotope dilution methods. It is evident from the results of quality control samples of MYCO-1 that isotope dilution strategies provide a more accurate quantitation than external calibration. Figure 4 also confirmed that ID^2^MS and ID^5^MS produce equivalent results. Moreover, the accuracy of these methods was confirmed, as they produced expected mass fractions for OTA in the quality control sample MYCO-1. However, the mass fractions of OTA obtained by ID^1^MS were on average 6% lower than those obtained by ID^2^MS and ID^5^MS for all samples (Table 2 and Fig. 4). This difference was attributed to isotopic enrichment bias [43–45] of the [^13^C_6_]-OTA relative to native OTA.

**Fig. 5:**
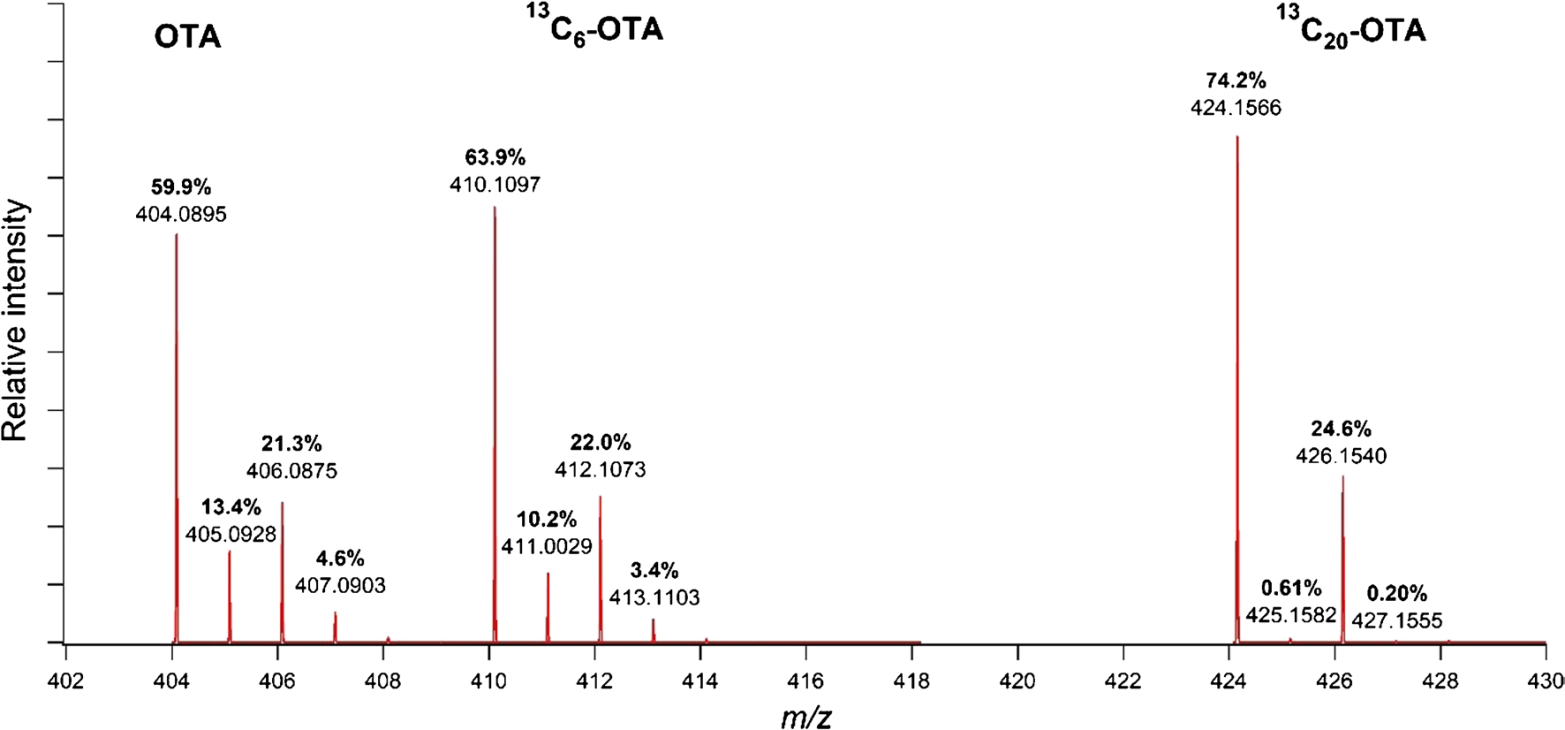
Isotopic pattern of native OTA, [^13^C_6_]-OTA, and [^13^C_20_]-OTA. The percent contribution of each isotopologue toward the full isotopic envelope is highlighted

Since the bias appeared systemic and ID^2^MS and ID^5^MS results were deemed accurate, gravimetric or other experimental sources of error were discarded. Instead, the bias originates from the fundamental principle of calculating isotopic abundance in high-resolution mass spectrometry based on the distribution of the monoisotopic mass (M) alone. The percent abundances of the monoisotopic mass (M) are not constant, but rather vary among unlabelled and their labelled counterparts. As shown in Fig. 5, the monoisotopic signal for unlabelled and [^13^C_6_]-OTA compromises 59.9% and 63.9% of their respective isotopic patterns. The isotope ratio (unlabelled:labelled) measured in the sample extracts is from the monoisotopic ions, specifically *m*/*z* 410.10967 for [^13^C_6_]-OTA which accounts for 63.9% of the isotopologues and *m*/*z* 404.08954 for native OTA given by 59.9% of the isotopologues. Therefore, the ID^1^MS results are expected to be biased by 6%, i.e., by a factor of 59.9/63.9.

It should be noted that this bias would be even more significant in the case of commonly used fully labelled [^13^C_20_]-OTA, where an increased monoisotopic contribution of 74.2% (Fig. 5) would result in a measurement bias of approximately 19% if a simple ID^1^MS calibration approach was employed. Additionally, it should be pointed out that the isotopic enrichment does not impact ID^n^MS. This is because the ratio of labelled to unlabelled OTA was measured in both the sample and calibration standard solutions separately and these two values cancel out in Eq. 2. The overall effect is that the direct measurement of the monoisotopic ion of the labelled internal standard ([^13^C_6_]-OTA) cancelled out and had no bearing in the final calculations. Therefore, when ID^1^MS is chosen as the quantitation method, one must be aware of the limitations of using the internal standard as the calibrator.

## Conclusions

In summary, four different calibration strategies were employed to determine the mass fraction of OTA in Canada Western Red Spring (CWRS) and Canada Western Amber Durum (CWAD) wheat. External standard calibration under-represented the mass fraction of OTA which was evident with QC samples of MYCO-1 which were 18–38% lower than the certified value. Ion suppression was clearly apparent and OTA mass fractions skewed lower than the expected values. External calibration is, therefore, an inaccurate quantitation method. Isotope dilution methods overcome matrix effects by instead measuring a ratio of the two isotopic forms of OTA.

ID^1^MS was the simplest quantitation method requiring a single sample which was spiked with a known amount of internal standard. For ID^1^MS, the exact concentration of the isotopically enriched standard must be known with confidence. In our study, the results obtained by ID^1^MS were found to be on average 6% lower than those obtained by higher order isotope dilution methods due to an apparent isotopic enrichment bias. This bias is more pronounced when the internal standard is highly labelled such as the case with [^13^C_20_]-OTA versus [^13^C_6_]-OTA.

OTAL-1 is a CRM with a certified mass fraction of [^13^C_6_]-OTA which fulfilled the requirements for ID^1^MS, but for many analytes an appropriate labelled reference material is unavailable. If this is the case, then ID^2^MS is the next best approach. A calibration standard solution consisting of an unlabelled reference standard and its labelled counterpart as the internal standard was prepared along with the sample extracts. The isotopic peak area ratios of both were measured and the known amount of unlabelled reference standard in the calibration standard solution was used to assign a mass fraction to the sample. This approach works best when the ratios of the sample and calibration standard solution are both close to 1 (exact-matching double isotope dilution). Multiple iterations are required to obtain the ideal ratio and a unique calibration standard solution is required for each sample making this method unrealistic for high throughput.

The final approach used for the quantitation of OTA was multi-point calibration with internal standard, specifically ID^5^MS. This method may require a bit more sample preparation upfront as multiple calibration standard solutions are prepared with varying isotopic ratios. However, for samples where the levels of OTA are unknown and differ between samples, this method is ideal. The calibration range can be tailored appropriately with little preliminary work required, maximizing throughput.
